# Lateral rectus muscle disinsertion and reattachment to the lateral orbital wall in exotropic Duane syndrome: a case report

**DOI:** 10.1186/1752-1947-2-253

**Published:** 2008-07-28

**Authors:** Dima Andalib, Alireza Javadzadeh

**Affiliations:** 1 Department of Ophthalmology, Nikookari Eye Hospital, Tabriz University of Medical Sciences, Tabriz, Iran

## Abstract

**Introduction:**

The surgical correction of anomalous movement such as upshoot in Duane syndrome is challenging. Lateral rectus muscle disinsertion and reattachment to the lateral orbital wall is a new approach used to minimize or eliminate the effects of co-contraction including globe retraction, palpebral fissure narrowing and anomalous vertical movement.

**Case presentation:**

We report a case of a 7-year-old boy who underwent this procedure for severe upshoot, globe retraction and exotropia in the left eye due to Duane syndrome. The patient achieved satisfactory ocular alignment following surgery. Upshoot and globe retraction were substantially improved.

**Conclusion:**

Lateral rectus muscle disinsertion and reattachment to the lateral orbital wall is a safe and effective procedure for weakening of the anomalous lateral rectus muscle in Exotropic Duane Syndrome.

## Introduction

Duane syndrome is an ocular motility disorder characterized by anomalous innervation of the lateral rectus muscle [1]. Abnormal innervation of the lateral rectus results in limitation to adduction and abduction, cocontraction of the horizontal rectus muscle, globe retraction, eyelid fissure changes and anomalous vertical movement [2], such as upshoot or downshoot [3]. Patients with Duane syndrome may have strabismus in the primary position, most commonly esotropia, and adopt a face turn to balance the alignment [1]. The upshoot or downshoot in Duane syndrome can be cosmetically unacceptable.

Various surgical approaches have been described for the treatment of upshoot and downshoot in Duane syndrome including recession of the lateral and medial rectus muscle, Y splitting of the lateral rectus muscle at the insertion, and posterior fixation suture of the horizontal rectus muscles [3]. Lateral rectus muscle disinsertion and reattachment to the lateral orbital wall is a new weakening procedure for special forms of strabismus including third nerve palsy and Duane syndrome with esotropia [4].

We report a case of a patient with unilateral Duane syndrome with exotropia who underwent this procedure for correction of severe upshoot and globe retraction.

## Case presentation

The parents of a 7-year-old boy noted that he had been unable to move his left eye outward since childhood. He had one strabismus surgery (large lateral rectus recession) in the left eye due to exotropia at the age of 3 years. Visual acuity was 20/20 in each eye. A 15° right face turn was found with 30 prism diopters' (PD) left exotropia. Versions revealed an inability to abduct the left eye with moderate limitation in adduction. He had significant upshoot and globe retraction in the right gaze. A left Exotropic Duane Syndrome with severe cocontraction was diagnosed.

**Figure 1:**
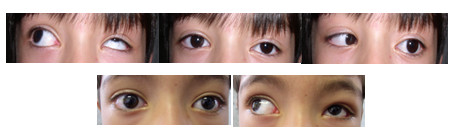
**Pre-operative (top) and 1-year postoperative (bottom) alignment in a patient with Exotropic Duane Syndrome in the left eye**. There was a significant improvement in upshoot after surgery.

At the time of surgery, intra-operative forced ductions revealed moderate restriction to adduction. A lateral limbal conjunctival incision was performed and the lateral rectus muscle was isolated on a muscle hook. The insertion of the lateral rectus muscle was in 17 mm posterior to the limbus. Blunt dissection between the lateral rectus and lateral orbital wall was performed and exposed the lateral periosteum 4 to 5 mm posterior to the orbital rim. Single, double-armed, 5-0 nonabsorbable polyester sutures (Ethicon) were placed and locked through the insertional end of the muscle. The lateral rectus muscle was disinserted and reattached to the periosteum with two bites. Intra-operative forced ductions were repeated at the end of surgery and revealed improvement in adduction (free passive adduction of the globe). Follow-up 1 year after surgery showed 6 PD of exotropia in the primary position, and a marked decrease in globe retraction, upshoot and face turn (Figure 1).

## Discussion

Exotropic Duane syndrome occurs with or without upshoot and/or downshoots and globe retraction in moderate exodeviations [5].

There are several theories for the anomalous vertical movements in Duane syndrome: compensatory oblique overaction, compensatory overaction of the vertical rectus muscles for defective abductive action [6], paradoxical synergistic innervations between the medial rectus muscle and superior rectus muscle [7] and the Bridle effect (cocontracting horizontal recti muscles) [6]. Electromyographic studies in patients with Duane syndrome have shown paradoxical innervation of the lateral rectus muscle in adduction, which results in a taut lateral rectus muscle in attempted adduction. The taut lateral rectus muscle slips sideways over the globe (bridle or leash phenomenon) and produces an anomalous vertical movement of the eye [3].

Posterior fixation sutures on the lateral rectus muscle for the stabilization of the muscle, recession of both medial rectus and lateral rectus, lowering of insertion of the lateral rectus muscle [3] and Y splitting of the lateral rectus muscle with recession are the current procedures for the correction of anomalous eye movement in Duane syndrome [5]. In Y splitting, the bifurcation of the muscle decreases upward or downward rotation of the globe because the halves are positioned to stabilize the muscle's position on the eye. Rao et al. [3] reported a significant decrease in the upshoot or downshoot in patients with Duane syndrome by Y splitting of the lateral rectus muscle with recession. In this report, the lateral rectus muscle was recessed 5 to 9 mm [3]. However, it is necessary to consider the primary position deviation when deciding on surgical procedures.

In Exotropic Duane Syndrome, the surgical goal is to correct the deviation and head turn and ameliorate as much of the anomalous eye movement as possible [5]. Surgical treatment frequently requires a large recession of anomalous lateral rectus muscle in an attempt to maintain alignment in the primary position and reduce the effects of misinnervation [4]. However, it is difficult to perform enough surgical weakening of the lateral rectus even with recession far back from the equator and suturing on the globe [5]. In a severe form of lateral rectus cocontraction, a large recession cannot eliminate the action of the anomalous firing lateral rectus muscle; therefore, surgery usually results in persistence as a recurrence of the effects of miswiring [4]. In our case, a large recession of the lateral rectus could not improve the anomalous eye movement.

Inactivating the muscle may decrease or eliminate the mechanical effects of the aberrant innervations [2]. Sato et al performed free myectomy of the lateral rectus in a subject with a third nerve palsy. Postoperative magnetic resonance imaging showed a contracting lateral rectus muscle attached to the globe by a fibrous tissue able to produce some abduction [8].

The lateral rectus muscle may be functionally inactivated by disinsertion and reattachment to the orbital periosteum. Britt et al. [2] reported three patients with esotropic Duane syndrome and marked globe retraction who underwent lateral rectus fixation to the orbital wall periosteum and partial vertical rectus muscles transposition augmented with posterior fixation sutures (Foster suture). In this report, all patients showed a significant improvement in the angle of deviation and globe retraction [2].

The advantages of this procedure over extirpation and free tenotomy include permanent disinsertion of the muscle from the globe and the ability to reverse the procedure if necessary. Suturing the rectus muscle to the orbital wall also reduces the risk of globe perforation compared with maximal recession. It may be technically difficult to expose the adjacent periosteum [4].

Our patient had recurrent exotropia with severe upshoot and globe retraction. We performed disinsertion of the lateral rectus muscle and reattachment to the orbital wall. It seems that the permanent lateral rectus muscle inactivation could eliminate the anomalous lateral rectus muscle; therefore, the upshoot and globe retraction were substantially decreased.

There is a residual abduction force due to periocular connective tissue contracture after longstanding deviation. The other cause is the abducting action of the oblique muscle [4]. Therefore, the elimination of the anomalous lateral rectus and improvement of adduction could not cause consecutive esotropia in the primary position in our patient.

## Conclusion

Lateral rectus muscle disinsertion and reattachment to the lateral orbital wall in a patient with Exotropic Duane Syndrome is an effective and stable procedure to improve ocular alignment, significant upshoot and severe globe retraction.

## Abbreviations

PD: Prism diopter.

## Competing interests

The authors declare that they have no competing interests.

## Authors' contributions

DA was responsible for the concept and wrote the paper. AJ reviewed and edited the manuscript. Both authors approved the final version.

## Consent

Written informed consent was obtained from the patient's next-of-kin for publication of this case report and any accompanying images. A copy of the written consent is available for review by the Editor-in-Chief of this journal.
